# Radiomics Modeling of Catastrophic Proximal Sesamoid Bone Fractures in Thoroughbred Racehorses Using μCT

**DOI:** 10.3390/ani12213033

**Published:** 2022-11-04

**Authors:** Parminder S. Basran, Sean McDonough, Scott Palmer, Heidi L. Reesink

**Affiliations:** 1 Clinical Sciences, Cornell University, Ithaca, NY 14853, USA; 2 Biomedical Sciences, Cornell University, Ithaca, NY 14853, USA; 3 Population Medicine and Diagnostic Sciences, Cornell University, Ithaca, NY 14853, USA; 4 Clinical Sciences, Equine and Farm Animal Hospital & Population Medicine and Diagnostic Sciences, Cornell University, Ithaca, NY 14853, USA

**Keywords:** equine, machine learning, computed tomography, Thoroughbred, fetlock

## Abstract

**Simple Summary:**

Mitigating the risk of catastrophic injuries in the horse racing industry remains a challenge. Non-invasive methods such as CT imaging in combination with machine learning could be used to screen horses at risk of injury, but there remain questions on the feasibility of such an approach. In this work, we investigated whether machine learning models could be developed from in vitro harvested μCT images of intact proximal sesamoid bones to predict whether the bone was from a horse that suffered a catastrophic injury or from a control group. The average accuracy in differentiating whether a sesamoid bone came from a case or control horse using our approach was 0.754. Our work suggests it may be possible to develop similar models using CT images of horses in the clinical setting.

**Abstract:**

Proximal sesamoid bone (PSB) fractures are the most common musculoskeletal injury in race-horses. X-ray CT imaging can detect expressed radiological features in horses that experienced catastrophic fractures. Our objective was to assess whether expressed radiomic features in the PSBs of 50 horses can be used to develop machine learning models for predicting PSB fractures. The μCTs of intact contralateral PSBs from 50 horses, 30 of which suffered catastrophic fractures, and 20 controls were studied. From the 129 intact μCT images of PSBs, 102 radiomic features were computed using a variety of voxel resampling dimensions. Decision Trees and Wrapper methods were used to identify the 20 top expressed features, and six machine learning algorithms were developed to model the risk of fracture. The accuracy of all machine learning models ranged from 0.643 to 0.903 with an average of 0.754. On average, Support Vector Machine, Random Forest (RUS Boost), and Log-regression models had higher performance than K-means Nearest Neighbor, Neural Network, and Random Forest (Bagged Trees) models. Model accuracy peaked at 0.5 mm and decreased substantially when the resampling resolution was greater than or equal to 1 mm. We find that, for this in vitro dataset, it is possible to differentiate between unfractured PSBs from case and control horses using μCT images. It may be possible to extend these findings to the assessment of fracture risk in standing horses.

## 1. Introduction

In the horseracing industry, there is a strong incentive to mitigate the risk of catastrophic peripheral bone fractures in Thoroughbred racehorses [1]. Upwards of 70% of Thoroughbred racing fatalities can be attributed to musculoskeletal injuries [2]. Catastrophic injuries are often observed as bone fractures in the forelimb fetlock joint, commonly affecting the proximal sesamoid bones (PSBs) and third metacarpal bone [3,4]. Several epidemiological factors, morphological, and radiological features have been associated with such fractures [5,6]. 

To date, there are no image-based screening modalities that could be used to identify Thoroughbred horses at risk of PSB fracture. Conventional CT could be used but acquiring standing scans has several challenges. Obtaining CT images of the horses under load bearing conditions is challenging as general anesthesia is required for compliance and to minimize the risk of motion artifacts. Some technical advances in standing CT have been suggested but whether these devices can achieve sufficient spatial resolution for diagnosis, mitigate the risk of motion and beam hardening artifacts, and whether these devices could be used in a screening capacity in the horseracing environment remains to be seen [7]. There have been similar advancements in standing MRI and PET technologies, but operationalizing them as a screening tool in the clinical and commercial setting remains a challenge [7,8,9].

There has been however substantial progress in using in vitro imaging in the equine setting to identify candidate image biomarkers for modeling the risk of fracture. Sub-millimeter resolution of PSBs can reveal exquisite morphological and structural details of the PSBs [10,11]. Studies exploring the use of micro-CT (μCT) of PSBs report radiological feature differences in the PSBs between horses that suffered catastrophic injuries with those that have not [10,11]. Recently, radiomics has been used as a strategy for computing image features from μCTs [12]. In this approach, hundreds of complex morphologic and texture features, often imperceptible by the human eye, can reveal features that correlate with underlying pathophysiology [13,14]. A promising aspect of radiomics is the observation that when radiomic calculation settings are chosen wisely, texture feature estimates are less sensitive to variations in image acquisition settings and modalities [15].

There has been much growth in the use of artificial intelligence in human and veterinary medicine over the last decade [16,17]. While these approaches may seem daunting to those less familiar with machine learning (ML), fundamentally, these methods can be broadly categorized as supervised and unsupervised ML. Supervised ML, also referred to as structured prediction, is where data is classified (e.g., fracture or no fracture) or a number is predicted. The supervised ML problem may be simply viewed as a regression or classification problem. There are many supervised ML methods to address the classification problem, in addition to the traditional Log-regression approach. Some important classification methods include Decision Trees, Support Vector Machines, K-means Nearest Neighbors, Random Forest, and Neural Networks [18,19]. The performance of different supervised ML methods can depend on the data analyzed. Unsupervised machine learning seeks to determine patterns within unlabeled data for inference. It is particularly useful in analyzing large datasets where associations between variables are unknown [20]. Semi-supervised machine learning is a combination of supervised and unsupervised learning and is often used when only few datasets are labelled.

Our interest is in developing artificial intelligence methods for the purpose of mitigating the risk of catastrophic fractures in horses. Our goal is to apply machine learning models from in vivo CT images of the horse fetlock. We hypothesize that machine learning models based on radiomic features from retrospectively collected in vitro μCT data can discriminate PSBs from horses that suffered catastrophic injury from those that did not. The purpose of this work was to investigate the performance of radiomics-based machine learning models to predict fracture based on μCT s of intact PSBs from Thoroughbred racehorses. 

## 2. Materials and Methods

Our study design was a retrospective one that uses μCT images of intact PSBs from cadavers of racehorses, used ML methods to seek discriminating μCT features that separated racehorses that suffered catastrophic fractures from those that did not, and applied machine learning methods to retrospectively model the risk of fracture from expressed features.

### 2.1. Image Datasets and Segmentations

**Figure 1:**
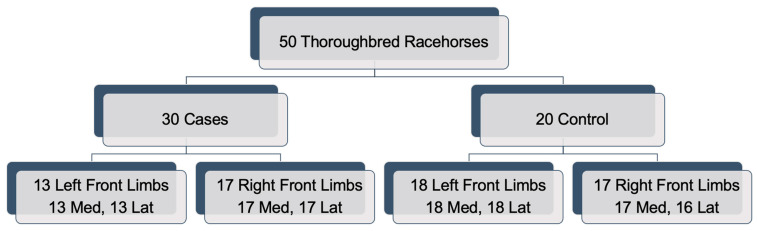
μCT datasets analyzed in this study. “Med” and “Lat” refer to the medial and lateral proximal sesamoid bones in the forelimbs.

Micro-CTs of the PSBs from 50 Thoroughbred horses were ethically obtained with permission from the New York State Gaming Commission. These racehorses were subjected to euthanasia or died on New York racetracks either from PSB fracture (cases) or from another injury not related to PSB fracture (controls). Horses underwent necroscopy within 72 h of death, and after necroscopy, PSBs were dissected, stored in saline-soaked gauze and frozen at −80 °C prior to μCT imaging. Samples were stored at −80 °C for 1–3 months prior to μCT imaging. Causes of death in the controls included cardiovascular collapse, colic, spinal fracture, pulmonary hemorrhage, and racing accidents. The median age of the horses was 4, ranging from 2 to 11 years. There were 23 females, 20 castrated males, and 7 intact males. Of the 50 horses, 30 suffered catastrophic fracture(s) of the PSBs in one forelimb, and 20 controls did not sustain any forelimb fetlock fracture (Figure 1). The PSBs from contralateral forelimbs from the horses that sustained fractures were defined as cases, whereas the controls consisted of the PSBs from both right and left forelimbs. When possible, the PSBs from both limbs from the control arm were scanned, but not all limbs nor PSBs were scanned and analyzed. One PSB in the control group was imaged but the image dataset was corrupted and thus excluded. Within the case group, 13 left and 17 right limbs had 2 intact PSBs scanned, and within the control group, 18 left and 17 right limbs had 1 or more intact PSBs scanned. A total of 129 intact PSBs were analyzed using a μCT scanner described in an earlier study [10]. Briefly, images were collected using high resolution micro-computed tomography with an isotropic voxel size of 50 μm, 720 projections, 20 ms exposure time, 100 kV, 50 mA (Zeiss Xradioa-520, Carl Zeiss Medica, Dublin, OH, USA). Most of the datasets were imaged with an isotropic resolution of 0.05 mm. A total of 31 and 34 left and right forelimbs were scanned, respectively, and 34 right medial, 33 right lateral, 31 left medial, and 31 left lateral PSBs were scanned. The μCT images were segmented using a simple 3D region-growing code based on an initial seed point within the sesamoid bone and provided a contiguous region of interest of the high-density bone tissue [21,22]. This region-growing code produced a binary file that identified the 3D extent of the high-density bone for radiomics analysis.

### 2.2. Image Biomarker Calculations

The PyRadiomics image informatics package (V3.0.1, Numpy 1.19.1, SimpleITK 2.0.0, PyWavelet 1.1.1, Python 3.6.7) was used in this study to compute radiomic features [23]. The ‘default’ settings were used to compute all image features. The total number of bins was fixed at 25 (FBS), a symmetrical grey level co-occurrence matrix was enforced, and no LoG kernel smoothing filter was applied. Radiomic features were computed with image data resampled over 0.075, 0.10, 0.25, 0.50, 1.00, and 2.00 mm. While numerous wavelet features were also computed, we analyzed 102 radiomic features as potential modeling parameters (Table 1). Radiomics platform performance was benchmarked with the Image Biomarker Standardization Initiative (IBSI) datasets [24]. All features were normalized using z-scale normalization prior to modeling. When examining individual feature differences between the cases and controls, Students’ T-test was used using the Matlab Statistical Analysis Package (R2021a, 9.10).

**Figure 2:**
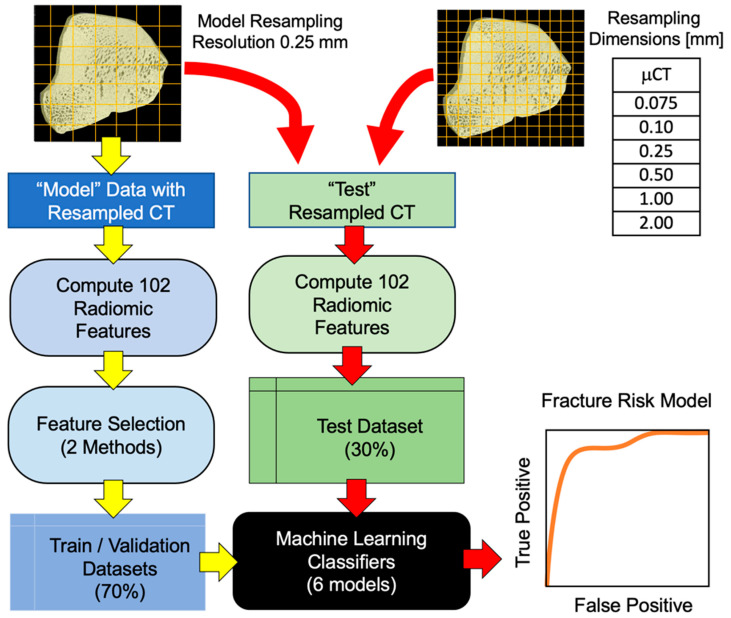
Experimental design of the μCT radiomics-based fracture risk modeling study. After segmenting the PSBs in the μCT, 102 radiomic features were computed using 6 different resampling dimensions. A resampling resolution of 0.25 mm was used as baseline data from which the top 3, 5, 10 and 20 expressed features were selected (using 2 different feature selection methods). Then, 6 classification models were trained and validated using these features, and the performance of these models were tested and ranked based on their accuracy. Finally, the top performing models, which were developed using 0.25 mm resolution radiomics data, were tested with the same PSB radiomics data using different resampling dimensions.

The utility of such a model is limited to μCT PSB data in the in vitro setting, which is not practical in the clinical setting. However, the μCT data can be resampled to larger sub-volumes such that the new voxel dimensions are comparable to those seen in conventional CT (Figure 2). Earlier work suggests feature differences from μCT PSBs are highly expressed when images are resampled in the range of 0.05 to 1.0 mm; thus, we chose 0.25 mm resolution data for feature selection and to develop baseline models (more extensive modeling with using different resampling dimensions for baseline models is available upon request). 

### 2.3. Feature Selection 

From the 129 datasets containing 102 features, only a subset of those features was used for modeling. Our feature selection process consisted of 2 approaches: a feature importance estimation method based on an ensemble of Decision Trees (DT) and a Wrapper Method (WR) [25]. In the first approach, we sought features which separated cases from controls using Decision Trees: if the value of a particular feature was above or below a threshold, data was separated into a new branch. This process was repeated until all data was classified. Each feature of the 102 features was assigned an importance factor proportional to its’ discriminating potential. Feature importance was obtained by training an ensemble of 100 classification trees followed by an ‘out-of-bag’ predictor importance estimate. To select features for modeling fracture risk, we simply selected the top 3, 5, 10 and 20 features based on the predictor importance score. The second feature selection approach, Wrapper Method, selects subsets of all the features that are best predictors and sequentially selects features until no improvement in the prediction is observed. The best predictor from the 102 features is found and a new feature that provides the best performance is sequentially added. From this, the top 3, 5, 10 and 20 features were then used for modeling. 

### 2.4. Supervised Machine Learning Models

Datasets were randomly partitioned into training/validation and testing datasets with a 70:30 ratio (90 training/validation and 39 independent test datasets). Six common classification algorithms were examined: logistic regression (LR), quadratic support vector machines (SVM), a k-nearest neighbor classifier (KNN), a ‘Bagged Trees’ ensemble (BT), a RUSBoosted ensemble (RUS), and a medium neural network (NN). Details of each model settings are provided in Table 2. Each of the models were trained (with training and validation data), and accuracy of the model when subjected to the test data was recorded. Accuracy is defined here as the fraction of correct predictions, where 1.000 is a perfect prediction of whether the test PSB features are from a case or control and 0.500 is (in this binary classification problem), a random guess between a case or a control.

After creating a model, we then subjected it to the (modelled) features computed from radiomics calculations when using a different resampling resolution (Figure 2). The top 3 performing models when using 3, 5, 10 and 20 features were selected, and the accuracy was recorded. Modeling was performed using the Matlab machine learning package (R2021a, 9.10). All calculations were performed on an iMac 10.15.7 and iMac Pro 11.2.1 both with 32 GB ram. 10-fold cross validation was performed to ensure random sampling effects did not influence estimates of accuracy.

## 3. Results

### 3.1. Top 20 Features

Table 3 displays the results of the top 20 features for the DT and WR methods using the radiomics data obtained from image data resampled at 0.25 mm. Of the top 20 discriminating features of the DT method, 5 were morphology features of the PSB (Sphericity; Surface to Volume ratio; Flatness, which is the ratio of the 2nd and 3rd principal component distances; Surface Area; and Least Axis), 5 were statistical or histogram features (Histogram Skewness; Root Mean Square; Mean; Median, and Total Energy), and the remaining were texture features. Of the top 20 features from the WR method, 1 was a morphology feature (Sphericity), 4 were statistical or histogram features (Mean Absolute Deviation; Variance, Maximum; and 90th Percentile of the Histogram), and the remaining 15 were texture features. The 4 common features from both DT and EM methods included 1 shape (Sphericity) and 3 texture features (GLCM Correlation; GLCM Cluster Shade; and GLCM lmc1).

### 3.2. Model Performance Using Decision Tree and Wrapper Method Features

Table 4 displays the accuracies from the 6 models when using either the top 3, 5, 10 and 20 DT or WR features. The LR model using 20 features from the DT feature selection method achieved the highest overall accuracy (0.903), and the KNN model using 5 features from the WR feature selection method produced the lowest accuracy (0.643). When using DT features, performance increased as N increased for all models except for the BT model. When comparing models irrespective of the number of features used in the model, the average accuracy of the LR model was highest (0.850), and SVM the lowest (0.728). Over all models and features selected, the average performance when using DT features was 0.774. 

**Figure 3:**
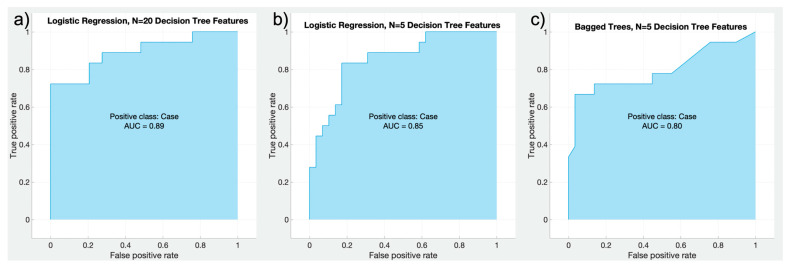
Three models of fracture risk from μCT images of PSBs tested on 39 datasets, trained from 90 testing and validation datasets. These models are: (**a**) a log-regression model using the top 20 features from a Decision Tree (DT) feature selection process (Table 3), (**b**) a similar log regression model with 5 DT features, and (**c**) a random forest Bagged Trees model with 10 DT features.

The LR model using 10 features from the WR feature selection achieved the highest overall accuracy (0.839), and the KNN model using 5 features from the WR feature selection produced the lowest accuracy (0.643). When using WR features, performance increased in the LR and SVM models as N decreased. When comparing models irrespective of feature used in the model, the average accuracy of the LR model was highest (0.826), and NN the lowest (0.692). Over all models and features selected, the average performance when using WR features was 0.734. Figure 3 displays the overall top 3 performing models along with their associated Area Under the Curve (AUC) values. 

### 3.3. Model Performance with Variable Voxel Resampling Dimensions

Again, the 129 datasets were divided into 90 training and validation and 39 test datasets. The accuracy of the (0.25 mm) models subjected to the resampled test data are displayed in Table 5. The overall accuracy of all models ranged from 0.602 (WR-RUS, N = 10) to 0.916 (DT-SVM, N = 20), with an average of 0.786 (95% Confidence Interval 0.675–0.892). On average, as the resampling resolution decreased, model performance remained relatively stable, but accuracy decreased as resampling resolution was 2.00 mm. Of all models and feature selection methods, SVM, RUS, and LR models had the highest number of top 3 performing models (7, 6 and 6 out of 24, respectively), and NN, BT, and KNN models had the lowest (2, 2, and 1, respectively).

When analyzing the resampling dimensions irrespective of the number of features modeled, feature selection method, or model, the average accuracy when using 0.075, 0.10, 0.50, 1.00, and 2.00 mm was 0.772, 0.801, 0.831, 0.793, and 0.792, respectively. On average, the highest accuracy achieved was with a sampling dimension of 0.50 mm. 

## 4. Discussion

This work suggests that machine learning models can differentiate the cases and controls, as recruited in this study, with relatively high accuracy (above 0.800) and with relatively few model parameters (from 3 to 20). Model performance depended on the resampling dimensions of the μCT data, the type of ML model deployed, the strategy for feature selection, and the number of features modeled.

Expressed features found in this study agree with some of our earlier findings which found PSB Width (or Least Axis), of cases and controls were statistically different [10,21]. The highest-ranking discriminating feature from both feature selection methods was Sphericity, which is defined as

$$Sphericity=\frac{\sqrt[{3}]{36\pi V^{2}}}{A}\;;$$

where *V* is the volume and *A* is the surface area. Sphericity is a measure of ‘roundness’ and ranges from 0 (flat) to 1 (sphere), and like many of the other highly expressed texture features is challenging to detect with the naked eye alone. Our study also observed increased mean μCT values (P = 0.014), which in combination with Sphericity suggests the PSBs in the cases are more compact, denser, and less spherical than the controls. Note that these two study groups were from the same group and thus similar findings may be the result of data sampled from similar populations. Similarities in radiomic feature differences were observed when using either the Python-based PyRadiomics package or the Matlab-based SERA package, which suggests that these feature differences are reproducible and may be less sensitive to inconspicuous settings when performing radiomics calculations.

Our baseline ML models were developed using μCT data resampled at a resolution of 0.25 mm, and when the models were subjected to image data resampled with resolution 1.00 mm or greater, model performance decreased substantially, and when data was resampled at resolutions of 0.5 mm, model performance peaked. For radiomics studies that have image datasets with a variety of pixel and voxel settings, or are collected from different imaging systems, resampling the image data can improve the robustness and reliability of feature calculations [26,27]. Pixel resolutions of 0.5 mm and smaller are achievable with modern standing equine CT imaging equipment; thus, imaging devices which can scan horse limbs in vivo at sub-millimeter resolutions may be preferable if using models developed in our study.

It is possible to stratify the datasets based on age, sex, or other variables such as medial versus lateral PSBs, and use conventional statistical methods (e.g., analysis of variance) to glean insights on candidate biomarkers. There is evidence medial PSBs undergo different forces than lateral PSBs and they fracture more commonly in unilateral fractures [3,28]. Exploring medial/lateral and other variables may be helpful from an epidemiological perspective; however, our goal was to test the methodology of ML approaches with image data exclusively, particularly since they can often out-perform standard (log-regression) modeling. Surprisingly, the log-regression models performed well when compared with more sophisticated ML approaches used in this study. As datasets increase in volume, variety, and velocity in the future, ML methods can offer computational efficiencies and offer decision support tools that are easy to understand and implement [29]. 

There are several limitations in our study. First, our study used feature differences in the contralateral and intact PSBs from racehorses that suffered catastrophic injury and compared them with intact PSBs of otherwise healthy racehorses within New York State. Features extracted from these two populations may not represent those observed in the broader horse-racing community. Whether radiomic features of the contralateral limb are representative of those in the fractured limb is challenging to determine [30,31]. Theoretically this could be assessed by examining the radiomic features of fractured PSBs and comparing them with the intact PSBs from horses that suffered catastrophic injuries. PSBs undergoing such injuries tend to fracture into many bone fragments, making the analysis of contiguous PSB bone tissue extremely challenging. In similar horses, bone volume fraction of PSBs appear to be associated with age at death, handicap rating, and age at first start of racing, but these variables were not examined in this study [11].

A second limitation stems from the fact that data was obtained from a μCT scanner under in vitro conditions. While all handling, processing, and imaging of the PSBs were consistent for all samples studied, there may be differences in CT features obtained in the in vivo versus in vitro conditions as the musculoskeletal system is under load and imaging equipment and technique would likely differ. As referenced earlier, resampling image data obtained from different imaging technologies help harmonize the radiomics data. Standardization of image quality and benchmark radiomics performance from different imaging technologies will be valuable if this approach is adopted using different imaging systems, populations, and communities.

There remains a need for efficient and non-invasive methods which can predict the risk of musculoskeletal injuries to manage catastrophic fractures and injuries, and monitor the health and training of horses to prevent such injuries [32]. This work suggests that radiomic features from resampled μCT data comparable to the voxel dimensions in conventional CT could be used in modeling the risk of fracture in PSBs. Other non-invasive imaging methods such as MRI, nuclear imaging, and infrared spectroscopy are able to detect lesions and abnormalities before the onset of gross disease [8,9,33,34,35,36]. Recently, a commercial option has been proposed for obtaining CT images in anesthetized standing horses [7]. Whether such systems can provide texture features of the PSBs comparable with μCT and whether those features could be used for modeling the risk of catastrophic injury remains in question.

One may argue that developing such models based on in vitro conditions may not be of value to the community. However, this work demonstrates that it is possible to develop machine learning models from μCT image data and outlines the approach for doing so. A prospective trial where high-resolution CT scans of Thoroughbred racehorses are obtained would be of value, but such an effort would require significant coordination between equine hospitals and the horseracing industry, and careful selection of study and control horses [31].

## 5. Conclusions

To conclude, we demonstrated machine learning models can differentiate the cases and controls recruited in this study with an average accuracy of 0.754, using relatively few model parameters (5 or greater). Adaptation of such models in the real-world setting where there is diversity in racehorse populations remains a topic of future research. 

## Figures and Tables

**Table 1:** Summary of the radiomic feature families and the specific features computed.

Feature Family	Feature Calculated
Morphology Shape (18)	Elongation, Flatness, Least Axis, Major Axis, Maximum 2&3D Diameter, Maximum 2D Row, Maximum 2DSlice, Mesh and Voxel Volume, Minor Axis Length, Sphericity, Surface Area, Surface to Volume Ratio
Statistics and Histogram First Order (18)	10th & 90th percentile, Energy, Entropy, Interquartile Range, Kurtosis, Maximum, Mean Absolute Deviation, Mean, Median, Minimum, Range, Robust Mean Absolute Deviation, Root Mean Squared, Skewness, Total Energy, Uniformity, Variance
Gray Level Co-occurrence Matrix GLCM Texture (24)	Autocorrelation, Cluster Prominence, Cluster Shade, Cluster Tendency, Contrast, Correlation, Difference Average, Difference Entropy, Difference Variance, LD, LDM, LDMN, LMC1, LMC2, Inverse Variance, Joint Average, Joint Entropy, Joint Energy, MCC, Maximum Probability, Sum Average, Sum Entropy, Sum Squares
Gray Level Difference Matrix GLDM Texture (14)	Dependence Entropy, Dependence Non-Uniformity +/− Normalized, Dependence Variance, Gray Level Non-Uniformity, Gray Level Variance, Large Dependence High/Low Gray Level Emphasis, Low Gray Level Emphasis, Small Dependence High/Low Gray Emphasis
Gray Level Run Length Matrix GLRLM Texture (16)	Gray level Non-Uniformity +/− Normalized, Gray level variance, High Gray Level Run Emphasis, Long Run Emphasis, Long Run High/Low Gray Level Emphasis, Run Entropy, Run Length Non-Uniformity +/− Normalized, Run Percentage, Run Variance, Short Run Emphasis, Short Run High Gray Level Emphasis
Gray Level Size Zone Matrix GLSZM Texture (16)	Short Run Low Gray Level Emphasis, Gray Level Non-Uniformity +/− Normalized, Gray Level Variance, High Gray Level Zone Emphasis, Large Area Emphasis, Large Area High/Low Gray Level Emphasis, Low Gray Level Zone Emphasis, Size Zone Non Uniformity +/− Normalized, Zone Entropy, Zone Percentage, Zone Variance

**Table 2:** Machine Learning models investigated and their associated descriptions and settings.

Model	Description and Model Settings
Logistic Regression (LR)	Conventional logistic regression model
Support Vector Machine (SVM)	Quadratic kernel, box constraint = 1, kernel scaling “auto”
K-nearest neighbor (KNN)	Medium Size, Number of Neighbors = 10, distance metric = Euclidean, Distance Weight = Equal
Ensemble—Bagged Trees (BT)	Learner Type: Decision Tree, maximum number of splits = 133, number of learners = 30
Ensemble—RUSBoosted Trees (RUS)	Learner Type: Decision Tree, maximum number of splits = 20, number of learners = 30, learning rate =0.1
Medium Neural Network (NN)	Number of layers = 1, First layer size = 25, Activation = ReLU, Iteration Limit = 1000, Regularization = off

**Table 3:** Top 20 radiomic features, in ascending order of priority, used in the ML models as generated from the Decision Tree (DT) classification, and Wrapper method (WR). Features with asterisk(*) were common in both DT and WR features.

Decision Trees (DT).	Wrapper Methods (WR)
Shape—Sphericity *	Shape—Sphericity *
Shape—Surface Volume Ratio	GLCM—Correlation *
GLSZM—Zone Entropy	GLSZM—Large Area Emphasis
SHAPE—Surface Area	First order—Mean Absolute Deviation
GLCM—Correlation *	First order—Variance
GLCM—Cluster Shade *	GLCM—Id
Shape—Flatness	First order—Maximum
GLCM—Imc1 *	GLCM—Idm
GLCM—Cluster Prominence	GLCM—Cluster Shade *
GLCM—Idmn	GLCM—Imc1 *
GLCM—Idn	GLCM—Inverse Variance
GLCM—Imc2	GLCM—Difference Entropy
GLSZM—Gray Level Variance	First order—90Percentile
First order—Skewness	GLRLM—Long Run Emphasis
GLSZM—Small Area Emphasis	GLRLM—Run Length Non-Uniformity Normalized
First order—RootMeanSquared	GLRLM—Run Percentage
First order—Median	GLRLM—Run Variance
First order—Mean	GLRLM—Short Run Emphasis
First order—Total Energy	GLSZM—Zone Percentage
Shape—Least Axis Length	GLRLM—Gray Level Variance

**Table 4:** Model performances for log-regression (LR), support vector machine (SVM), k-nearest neighbor (KNN), Bagged Trees (BT), RUSBoost Trees (RUS), and medium sized neural network (NN) using 0.25 mm resampling resolution.

Decision Tree Feature Model Accuracy				
	N = 3	N = 5	N = 10	N = 20
LR	0.793	0.856	0.847	0.903
SVM	0.681	0.702	0.734	0.797
KNN	0.674	0.686	0.787	0.792
BT	0.733	0.808	0.845	0.808
RUS	0.733	0.738	0.808	0.834
NN	0.680	0.749	0.792	0.787
**Wrapper Method Feature Model Accuracy**				
	N = 3	N = 5	N = 10	N = 20
LR	0.793	0.837	0.839	0.833
SVM	0.702	0.723	0.750	0.765
KNN	0.712	0.643	0.717	0.819
BT	0.734	0.734	0.712	0.701
RUS	0.728	0.717	0.728	0.669
NN	0.685	0.674	0.728	0.680

**Table 5:** Accuracies of top 3 models for 3, 5, 10, and 20 features from Table 3 and Table 4 when subjected to different resampling dimensions of the μCT data.

Number Features	Feature Selection	Model	Resampling Dimension [mm]				
			0.075	0.10	0.50	1.00	2.00
3	DT	SVM	0.829	0.850	0.872	0.858	0.829
		RUS	0.699	0.763	0.835	0.783	0.675
		KNN	0.733	0.756	0.821	0.779	0.746
	WR	SVM	0.842	0.854	0.870	0.851	0.806
		LR	0.713	0.750	0.733	0.683	0.619
		RUS	0.715	0.757	0.786	0.716	0.677
5	DT	BT	0.844	0.857	0.870	0.859	0.832
		LR	0.682	0.726	0.807	0.767	0.662
		RUS	0.681	0.703	0.805	0.743	0.692
	WR	SVM	0.843	0.853	0.869	0.857	0.817
		LR	0.711	0.732	0.768	0.731	0.695
		RUS	0.658	0.719	0.759	0.688	0.660
10	DT	SVM	0.880	0.897	0.892	0.858	0.805
		BT	0.834	0.850	0.844	0.775	0.739
		NN	0.718	0.772	0.839	0.781	0.684
	WR	SVM	0.849	0.857	0.867	0.861	0.837
		LR	0.747	0.768	0.775	0.753	0.711
		RUS	0.720	0.746	0.792	0.731	0.602
20	DT	SVM	0.899	0.916	0.901	0.863	0.808
		LR	0.784	0.811	0.908	0.898	0.787
		RUS	0.763	0.806	0.849	0.813	0.673
	WR	LR	0.849	0.856	0.867	0.864	0.838
		SVM	0.827	0.858	0.844	0.767	0.697
		NN	0.758	0.774	0.771	0.753	0.685

## Data Availability

Radiomics data and machine learning models are available upon request.
